# Potassium trinickel(II) orthophosphate diphosphate, KNi_3_(PO_4_)P_2_O_7_

**DOI:** 10.1107/S1600536813034089

**Published:** 2013-12-24

**Authors:** Meryem Moutataouia, Mohammed Lamire, Mohamed Saadi, Lahcen El Ammari

**Affiliations:** a Laboratoire de Physico-Chimie des Matériaux Inorganiques, Faculté des Sciences Aïn Chock, Casablanca, Morocco; b Laboratoire de Chimie du Solide Appliquée, Faculté des Sciences, Université Mohammed V-Agdal, Avenue Ibn Batouta, BP 1014, Rabat, Morocco

## Abstract

The structure of the title compound is characterized by the presence of two different anions, (PO_4_)^3−^ and (P_2_O_7_)^4−^ with an eclipsed conformation. The crystal structure consists of edge-sharing [NiO_6_] octa­hedra forming an [Ni_3_O_14_] chain running parallel to [001]. Adjacent chains are connected through edges and apices to PO_4_ and P_2_O_7_ groups in such a way as to build a three-dimensional host lattice. The resulting framework presents inter­secting tunnels running along [010] and [101] in which the 11-coordinated potassium cation is located. The crystal structure of this new phosphate probably represents a new structural type.

## Related literature   

For example of crystal structures with mixed phosphate anions, see: Ayed (2012 ▶); Palkina & Maksimova (1980 ▶); Nagornyi *et al.* (1996 ▶); Sanz *et al.* (1996 ▶, 1999 ▶, 2001 ▶).

## Experimental   

### 

#### Crystal data  

KNi_3_(PO_4_)P_2_O_7_
*M* *_r_* = 484.14
Monoclinic, 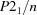

*a* = 9.8591 (3) Å
*b* = 9.3953 (3) Å
*c* = 9.9778 (3) Å
β = 118.965 (1)°
*V* = 808.63 (4) Å^3^
*Z* = 4
Mo *K*α radiation
μ = 8.09 mm^−1^
*T* = 296 K
0.25 × 0.18 × 0.12 mm

#### Data collection  

Bruker X8 APEX diffractometer
Absorption correction: multi-scan (*SADABS*; Bruker, 2009 ▶) *T*_min_ = 0.223, *T*_max_ = 0.443
14936 measured reflections
3543 independent reflections
3352 reflections with *I* > 2σ(*I*)
*R*_int_ = 0.036

#### Refinement  

*R*[*F* ^2^ > 2σ(*F* ^2^)] = 0.020
*wR*(*F* ^2^) = 0.050
*S* = 1.06
3543 reflections
164 parameters
Δρ_max_ = 0.69 e Å^−3^
Δρ_min_ = −0.91 e Å^−3^

### 

Data collection: *APEX2* (Bruker, 2009 ▶); cell refinement: *SAINT* (Bruker, 2009 ▶); data reduction: *SAINT*; program(s) used to solve structure: *SHELXS97* (Sheldrick, 2008 ▶); program(s) used to refine structure: *SHELXL97* (Sheldrick, 2008 ▶); molecular graphics: *ORTEP-3 for Windows* (Farrugia, 2012 ▶) and *DIAMOND* (Brandenburg, 2006 ▶); software used to prepare material for publication: *publCIF* (Westrip, 2010 ▶).

## Supplementary Material

Crystal structure: contains datablock(s) I. DOI: 10.1107/S1600536813034089/br2234sup1.cif

Structure factors: contains datablock(s) I. DOI: 10.1107/S1600536813034089/br2234Isup2.hkl

Additional supporting information:  crystallographic information; 3D view; checkCIF report

## Figures and Tables

**Fig. 1.**
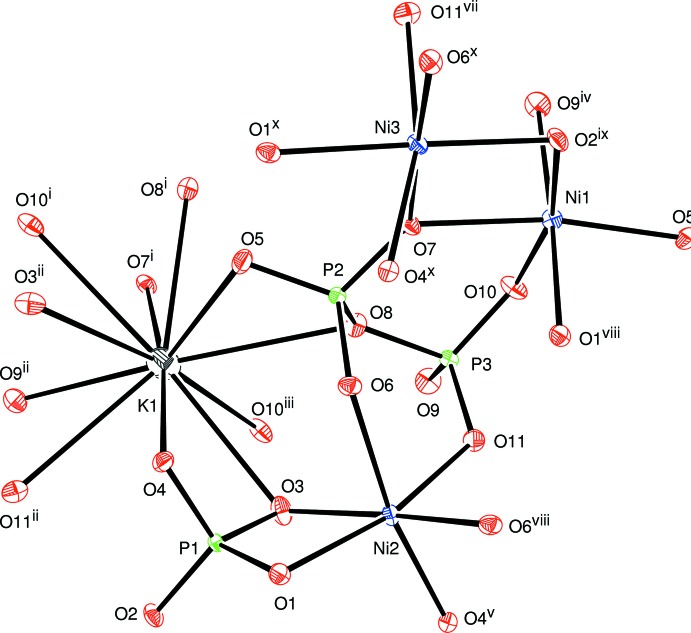
Plot of KNi3(PO4)P2O7 crystal structure showing polyhedra linkage. Displacement ellipsoids are drawn at the 70% probability level. Symmetry codes:(i) x + 1/2, -y + 1/2, z + 1/2; (ii) -x + 3/2, y - 1/2, -z + 3/2; (iii) x, y - 1, z; (iv) -x + 2, -y + 1, -z + 1; (v) x + 1/2, -y + 3/2, z + 1/2; (vi) x - 1/2, -y + 1/2, z - 1/2; (vii) -x + 3/2, y - 1/2, -z + 1/2; (viii) x - 1/2, -y + 3/2, z - 1/2; (ix) -x + 3/2, y + 1/2, -z + 3/2; (x) -x + 1, -y + 1, -z + 1.

**Fig. 2.**
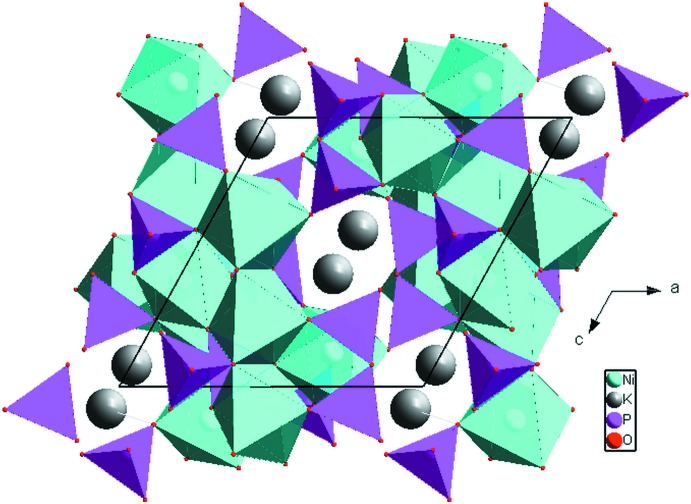
Projection views of the KNi3(PO4)P2O7 framework structure showing tunnels running along b directions where the potassium atoms are located.

## supplementary crystallographic information

### 1. Comment

Despite the large number of new structures of transition metal phosphates determined in recent years, only a limited number of phosphates show the existence of P_2_O_7_ diphosphate and PO_4_ monophosphate groups in their structures. Of these compounds, we report AgCr_2_(PO_4_)(P_2_O_7_) discovered by Ayed (2012). The K_2_Ni_4_(PO_4_)_2_(P_2_O_7_), Na_4_Ni_5_(PO_4_)_2_(P_2_O_7_)_2_ and Na_4_*M*^II^_3_(PO_4_)_2_(P_2_O_7_), (*M*^II^ = Mn, Co, Ni) compounds were respectively developed by Palkina & Maksimova (1980), Nagornyi *et al.* (1996) and Sanz *et al.* (1996, 1999 and 2001). In the present work, we report the synthesis and structural determination of the new mixed anion phosphate, KNi_3_(PO_4_)(P_2_O_7_), from single-crystal X-ray diffraction data.

The partial three-dimensional plot in Fig.1 shows the connection ion-oxygen polyhedra in the crystal structure of the title compound. In the PO_4_ tetrahedron, the P–O distances and O–P–O angles respectively between 1.5170—1.5916 (9) Å and 104.70—114.66 (5) Å are consistent with values found in the literature. In the P_2_O_7_ group, the values of the P–O distances are in the range of 1.4924—1.5659 (9) Å while the shared oxygen is at P2–O8–P3 between 1.5971—1.6408 (9) Å and the angle P2—O8—P3 = 125.38 (6)°. P_2_O_7_ adopts an eclipsed conformation as indicated by the dihedral angle of 7.42°, between the two plans through O5P2O8 and O8P3O9.

In this structure the three independent nickel atoms occupy regular octahedra with Ni–O distances between 2.0027 (9) Å and 2.2284 (9) Å. The [Ni1O_6_], [Ni2O_6_] and [Ni3O_6_] octahedra share edges and form a chain directed along the *c* axis. The chains are linked together by PO_4_ and P_2_O_7_ tetrahedra and by sharing two edges of two octahedra in the way to form a layer perpendicular to the *b* axis. The potassium atom K1, is coordinated to eleven oxygen atoms building a very distorted polyhedron.

The resulting 3-D framework presents intersecting tunnels running along the [010] and [001] directions, where the potassium cation is located (Fig.2). Probably, the structure of this phosphate represents a new structural type.

### 2. Experimental

KNi_3_(PO_4_)P_2_O_7_ is obtained during the preparation of NaK_5_Ni_4_Co(P_2_O_7_)_4_ diphosphate. The powder of this phosphate was prepared by solid state reaction, by mixing the nominal proportions reagents NaNO_3_, KNO_3_, Ni(NO_3_)_2_,6H_2_O, Co(NO_3_)_2_,6H_2_O and (NH_4_)H_2_PO_4_. The mixture is put into a platinum crucible, and subjected to thermal treatment (473 K, 673 K and 873 K) interspersed with grinding until a temperature of 1073 K. The final powder is brown colour.

The prepared powder by the solid route is gradually increased to a temperature above its melting point (1273 K) for 2 h, followed by slow cooling to about 5 K per hour to 673 K. Then, the power supply of the furnace is cut, and cooling is continued to room temperature. Single crystals of brown colour are obtained.

### 3. Refinement

The highest peak and the deepest hole in the final Fourier map are at 0.70 Å and 0.84 Å, respectively, from O3 and Ni2. The not significant bonds and angles were removed from the CIF file.

### Crystal data

**Table:**

KNi_3_(PO_4_)P_2_O_7_	*F*(000) = 944
*M**_r_* = 484.14	*D*_x_ = 3.977 Mg m^−^^3^
Monoclinic, *P*2_1_/*n*	Mo *K*α radiation, λ = 0.71073 Å
Hall symbol: -P 2yn	Cell parameters from 3543 reflections
*a* = 9.8591 (3) Å	θ = 3.2–35.0°
*b* = 9.3953 (3) Å	µ = 8.09 mm^−^^1^
*c* = 9.9778 (3) Å	*T* = 296 K
β = 118.965 (1)°	Block, brown
*V* = 808.63 (4) Å^3^	0.25 × 0.18 × 0.12 mm
*Z* = 4	

### Data collection

**Table:**

Bruker X8 APEX diffractometer	3543 independent reflections
Radiation source: fine-focus sealed tube	3352 reflections with *I* > 2σ(*I*)
Graphite monochromator	*R*_int_ = 0.036
φ and ω scans	θ_max_ = 35.0°, θ_min_ = 3.2°
Absorption correction: multi-scan (*SADABS*; Bruker, 2009)	*h* = −15→15
*T*_min_ = 0.223, *T*_max_ = 0.443	*k* = −12→15
14936 measured reflections	*l* = −15→16

### Refinement

**Table:**

Refinement on *F*^2^	Primary atom site location: structure-invariant direct methods
Least-squares matrix: full	Secondary atom site location: difference Fourier map
*R*[*F*^2^ > 2σ(*F*^2^)] = 0.020	*w* = 1/[σ^2^(*F*_o_^2^) + (0.021*P*)^2^ + 0.5342*P*] where *P* = (*F*_o_^2^ + 2*F*_c_^2^)/3
*wR*(*F*^2^) = 0.050	(Δ/σ)_max_ = 0.001
*S* = 1.06	Δρ_max_ = 0.69 e Å^−^^3^
3543 reflections	Δρ_min_ = −0.91 e Å^−^^3^
164 parameters	Extinction correction: *SHELXL97* (Sheldrick, 2008), Fc^*^=kFc[1+0.001xFc^2^λ^3^/sin(2θ)]^-1/4^
0 restraints	Extinction coefficient: 0.0100 (4)

### Special details

**Table:**

Geometry. All s.u.'s (except the s.u. in the dihedral angle between two l.s. planes) are estimated using the full covariance matrix. The cell s.u.'s are taken into account individually in the estimation of s.u.'s in distances, angles and torsion angles; correlations between s.u.'s in cell parameters are only used when they are defined by crystal symmetry. An approximate (isotropic) treatment of cell s.u.'s is used for estimating s.u.'s involving l.s. planes.
Refinement. Refinement of *F*^2^ against all reflections. The weighted *R*-factor *wR* and goodness of fit *S* are based on *F*^2^, conventional *R*-factors *R* are based on *F*, with *F* set to zero for negative *F*^2^. The threshold expression of *F*^2^ > 2σ(*F*^2^) is used only for calculating *R*-factors(gt) *etc*. and is not relevant to the choice of reflections for refinement. *R* factors based on *F*^2^ are statistically about twice as large as those based on *F*, and *R*- factors based on all data will be even larger.

### Fractional atomic coordinates and isotropic or equivalent isotropic displacement parameters (Å^2^)

**Table:**

	*x*	*y*	*z*	*U*_iso_*/*U*_eq_	
Ni1	0.861648 (18)	0.127830 (16)	0.624602 (19)	0.00491 (4)	
Ni2	1.007773 (18)	0.632051 (17)	0.603321 (18)	0.00441 (4)	
Ni3	0.679301 (17)	0.131207 (17)	0.264248 (18)	0.00438 (4)	
K1	0.50173 (3)	0.73539 (3)	0.41894 (4)	0.01315 (6)	
P1	0.82799 (3)	0.83779 (3)	0.43146 (3)	0.00345 (5)	
P2	0.69371 (3)	0.42611 (3)	0.42330 (3)	0.00344 (6)	
P3	0.85111 (3)	0.44065 (3)	0.75246 (3)	0.00384 (6)	
O1	0.96823 (10)	0.77074 (9)	0.41635 (10)	0.00519 (14)	
O2	0.82563 (10)	1.00027 (9)	0.43452 (10)	0.00565 (14)	
O3	0.84288 (10)	0.77158 (10)	0.57665 (10)	0.00698 (15)	
O4	0.68760 (10)	0.77531 (10)	0.28581 (10)	0.00575 (14)	
O5	0.54105 (10)	0.47889 (10)	0.30043 (10)	0.00712 (15)	
O6	0.83385 (10)	0.49624 (10)	0.41561 (10)	0.00516 (14)	
O7	0.70827 (10)	0.26402 (9)	0.43661 (10)	0.00567 (14)	
O8	0.70879 (10)	0.48003 (10)	0.58176 (10)	0.00553 (14)	
O9	0.81763 (10)	0.52456 (10)	0.86045 (10)	0.00722 (15)	
O10	0.85298 (11)	0.28110 (10)	0.76671 (11)	0.00720 (15)	
O11	0.99874 (10)	0.49156 (10)	0.74974 (10)	0.00609 (15)	

### Atomic displacement parameters (Å^2^)

**Table:**

	*U*^11^	*U*^22^	*U*^33^	*U*^12^	*U*^13^	*U*^23^
Ni1	0.00522 (7)	0.00507 (8)	0.00467 (7)	0.00009 (4)	0.00258 (6)	0.00000 (5)
Ni2	0.00475 (7)	0.00416 (7)	0.00444 (7)	0.00088 (4)	0.00232 (6)	0.00091 (4)
Ni3	0.00555 (7)	0.00355 (8)	0.00402 (7)	−0.00021 (4)	0.00230 (6)	0.00010 (4)
K1	0.01425 (12)	0.01237 (13)	0.01689 (13)	0.00032 (9)	0.01076 (10)	0.00072 (10)
P1	0.00407 (11)	0.00287 (12)	0.00361 (12)	0.00047 (8)	0.00202 (9)	0.00016 (9)
P2	0.00370 (11)	0.00332 (12)	0.00354 (12)	−0.00001 (8)	0.00194 (9)	−0.00022 (9)
P3	0.00463 (11)	0.00382 (12)	0.00401 (12)	−0.00023 (9)	0.00284 (9)	−0.00035 (9)
O1	0.0048 (3)	0.0046 (3)	0.0073 (4)	0.0006 (3)	0.0038 (3)	0.0003 (3)
O2	0.0076 (3)	0.0031 (4)	0.0053 (3)	0.0008 (3)	0.0025 (3)	0.0000 (3)
O3	0.0092 (4)	0.0073 (4)	0.0064 (4)	0.0025 (3)	0.0053 (3)	0.0023 (3)
O4	0.0045 (3)	0.0050 (4)	0.0061 (3)	0.0000 (3)	0.0012 (3)	−0.0008 (3)
O5	0.0053 (3)	0.0072 (4)	0.0058 (4)	0.0007 (3)	0.0003 (3)	−0.0002 (3)
O6	0.0049 (3)	0.0052 (4)	0.0065 (4)	−0.0007 (3)	0.0036 (3)	−0.0002 (3)
O7	0.0077 (3)	0.0034 (4)	0.0060 (4)	0.0002 (3)	0.0034 (3)	−0.0003 (3)
O8	0.0059 (3)	0.0067 (4)	0.0042 (3)	0.0007 (3)	0.0025 (3)	−0.0005 (3)
O9	0.0086 (4)	0.0084 (4)	0.0062 (4)	0.0006 (3)	0.0048 (3)	−0.0020 (3)
O10	0.0112 (4)	0.0042 (4)	0.0079 (4)	−0.0006 (3)	0.0060 (3)	−0.0005 (3)
O11	0.0052 (3)	0.0068 (4)	0.0074 (4)	0.0002 (3)	0.0039 (3)	0.0019 (3)

### Geometric parameters (Å, º)

**Table:**

Ni1—O5^i^	2.0509 (9)	K1—O5	2.7928 (10)
Ni1—O10	2.0515 (9)	K1—O8^x^	2.8966 (9)
Ni1—O9^ii^	2.0840 (9)	K1—O3	2.9627 (9)
Ni1—O2^iii^	2.1230 (9)	K1—O3^viii^	2.9901 (9)
Ni1—O1^iv^	2.1335 (9)	K1—O7^x^	3.0421 (9)
Ni1—O7	2.1668 (9)	K1—O8	3.0578 (9)
Ni2—O11	2.0027 (9)	K1—O11^viii^	3.0633 (10)
Ni2—O3	2.0033 (9)	K1—O10^x^	3.0677 (10)
Ni2—O4^v^	2.0223 (9)	P1—O3	1.5170 (9)
Ni2—O6^iv^	2.0524 (9)	P1—O2	1.5273 (9)
Ni2—O1	2.1508 (9)	P1—O4	1.5569 (9)
Ni2—O6	2.2284 (9)	P1—O1	1.5916 (9)
Ni3—O2^iii^	2.0266 (9)	P2—O5	1.4924 (9)
Ni3—O7	2.0299 (9)	P2—O7	1.5294 (9)
Ni3—O11^vi^	2.0666 (9)	P2—O6	1.5659 (9)
Ni3—O4^vii^	2.1069 (9)	P2—O8	1.5971 (9)
Ni3—O1^vii^	2.1314 (9)	P3—O9	1.4953 (9)
Ni3—O6^vii^	2.1494 (9)	P3—O10	1.5050 (10)
K1—O4	2.7605 (9)	P3—O11	1.5447 (9)
K1—O9^viii^	2.7742 (10)	P3—O8	1.6408 (9)
K1—O10^ix^	2.7790 (10)		
			
O5^i^—Ni1—O10	93.40 (4)	O8^x^—K1—O3	135.18 (3)
O5^i^—Ni1—O9^ii^	96.96 (4)	O4—K1—O3^viii^	63.64 (2)
O10—Ni1—O9^ii^	87.52 (4)	O9^viii^—K1—O3^viii^	81.38 (3)
O5^i^—Ni1—O2^iii^	100.96 (4)	O10^ix^—K1—O3^viii^	172.36 (3)
O10—Ni1—O2^iii^	165.64 (4)	O5—K1—O3^viii^	66.47 (3)
O9^ii^—Ni1—O2^iii^	91.17 (4)	O8^x^—K1—O3^viii^	90.21 (3)
O5^i^—Ni1—O1^iv^	87.13 (4)	O3—K1—O3^viii^	116.18 (3)
O10—Ni1—O1^iv^	96.81 (3)	O4—K1—O7^x^	172.06 (3)
O9^ii^—Ni1—O1^iv^	173.88 (4)	O9^viii^—K1—O7^x^	64.66 (3)
O2^iii^—Ni1—O1^iv^	83.57 (3)	O10^ix^—K1—O7^x^	64.03 (3)
O5^i^—Ni1—O7	168.65 (4)	O5—K1—O7^x^	117.91 (3)
O10—Ni1—O7	86.48 (4)	O8^x^—K1—O7^x^	49.54 (2)
O9^ii^—Ni1—O7	94.38 (3)	O3—K1—O7^x^	127.31 (3)
O2^iii^—Ni1—O7	79.36 (3)	O3^viii^—K1—O7^x^	116.17 (3)
O1^iv^—Ni1—O7	81.62 (3)	O4—K1—O8	86.23 (3)
O11—Ni2—O3	102.02 (4)	O9^viii^—K1—O8	161.82 (3)
O11—Ni2—O4^v^	87.49 (4)	O10^ix^—K1—O8	71.04 (3)
O3—Ni2—O4^v^	98.00 (4)	O5—K1—O8	49.77 (3)
O11—Ni2—O6^iv^	89.12 (4)	O8^x^—K1—O8	75.30 (3)
O3—Ni2—O6^iv^	167.61 (4)	O3—K1—O8	60.91 (2)
O4^v^—Ni2—O6^iv^	87.78 (4)	O3^viii^—K1—O8	115.80 (3)
O11—Ni2—O1	168.14 (3)	O7^x^—K1—O8	100.50 (2)
O3—Ni2—O1	72.17 (3)	O4—K1—O11^viii^	56.81 (3)
O4^v^—Ni2—O1	103.41 (4)	O9^viii^—K1—O11^viii^	51.13 (2)
O6^iv^—Ni2—O1	95.88 (3)	O10^ix^—K1—O11^viii^	110.78 (3)
O11—Ni2—O6	87.01 (4)	O5—K1—O11^viii^	117.10 (3)
O3—Ni2—O6	91.05 (4)	O8^x^—K1—O11^viii^	140.19 (3)
O4^v^—Ni2—O6	170.22 (4)	O3—K1—O11^viii^	84.38 (2)
O6^iv^—Ni2—O6	84.05 (4)	O3^viii^—K1—O11^viii^	61.90 (2)
O1—Ni2—O6	82.84 (3)	O7^x^—K1—O11^viii^	115.62 (3)
O2^iii^—Ni3—O7	84.95 (4)	O8—K1—O11^viii^	140.86 (2)
O2^iii^—Ni3—O11^vi^	87.67 (4)	O4—K1—O10^x^	123.00 (3)
O7—Ni3—O11^vi^	99.52 (4)	O9^viii^—K1—O10^x^	58.37 (3)
O2^iii^—Ni3—O4^vii^	108.45 (4)	O10^ix^—K1—O10^x^	120.20 (3)
O7—Ni3—O4^vii^	87.57 (4)	O5—K1—O10^x^	92.99 (3)
O11^vi^—Ni3—O4^vii^	163.00 (4)	O8^x^—K1—O10^x^	50.00 (2)
O2^iii^—Ni3—O1^vii^	178.03 (3)	O3—K1—O10^x^	174.53 (3)
O7—Ni3—O1^vii^	95.54 (4)	O3^viii^—K1—O10^x^	59.72 (2)
O11^vi^—Ni3—O1^vii^	94.13 (3)	O7^x^—K1—O10^x^	56.48 (2)
O4^vii^—Ni3—O1^vii^	69.68 (3)	O8—K1—O10^x^	123.62 (3)
O2^iii^—Ni3—O6^vii^	94.15 (3)	O11^viii^—K1—O10^x^	90.32 (3)
O7—Ni3—O6^vii^	175.46 (3)	O3—P1—O2	112.71 (5)
O11^vi^—Ni3—O6^vii^	84.87 (3)	O3—P1—O4	111.59 (5)
O4^vii^—Ni3—O6^vii^	88.50 (3)	O2—P1—O4	112.35 (5)
O1^vii^—Ni3—O6^vii^	85.21 (3)	O3—P1—O1	103.97 (5)
O4—K1—O9^viii^	107.91 (3)	O2—P1—O1	114.83 (5)
O4—K1—O10^ix^	115.00 (3)	O4—P1—O1	100.53 (5)
O9^viii^—K1—O10^ix^	92.23 (3)	O5—P2—O7	114.66 (5)
O4—K1—O5	69.69 (3)	O5—P2—O6	112.50 (5)
O9^viii^—K1—O5	145.41 (3)	O7—P2—O6	112.05 (5)
O10^ix^—K1—O5	120.66 (3)	O5—P2—O8	106.34 (5)
O4—K1—O8^x^	137.36 (3)	O7—P2—O8	105.67 (5)
O9^viii^—K1—O8^x^	99.93 (3)	O6—P2—O8	104.70 (5)
O10^ix^—K1—O8^x^	95.04 (3)	O9—P3—O10	117.00 (5)
O5—K1—O8^x^	69.04 (3)	O9—P3—O11	112.84 (5)
O4—K1—O3	52.60 (3)	O10—P3—O11	109.97 (5)
O9^viii^—K1—O3	118.50 (3)	O9—P3—O8	104.65 (5)
O10^ix^—K1—O3	63.30 (3)	O10—P3—O8	106.73 (5)
O5—K1—O3	88.24 (3)	O11—P3—O8	104.55 (5)

Symmetry codes: (i) *x*+1/2, −*y*+1/2, *z*+1/2; (ii) −*x*+3/2, *y*−1/2, −*z*+3/2; (iii) *x*, *y*−1, *z*; (iv) −*x*+2, −*y*+1, −*z*+1; (v) *x*+1/2, −*y*+3/2, *z*+1/2; (vi) *x*−1/2, −*y*+1/2, *z*−1/2; (vii) −*x*+3/2, *y*−1/2, −*z*+1/2; (viii) *x*−1/2, −*y*+3/2, *z*−1/2; (ix) −*x*+3/2, *y*+1/2, −*z*+3/2; (x) −*x*+1, −*y*+1, −*z*+1.
